# Phylogenetic Divergence and Molecular Signatures of Porcine Epidemic Diarrhea Virus S‐INDEL Subtype

**DOI:** 10.1155/tbed/1604677

**Published:** 2026-09-12

**Authors:** Yue Chen, Lin Yuan, Wenqiang Wang, Lulu Chen, Can Cui, Jie Zhang, Zhenbang Zhu, Wei Wen, Kegong Tian, Xiangdong Li

**Affiliations:** ^1^ Jiangsu Co-Innovation Center for Prevention and Control of Important Animal Infectious Diseases and Zoonoses, College of Veterinary Medicine, Yangzhou University, Yangzhou 225009, China, yzu.edu.cn; ^2^ Beijing Sino-Science Gene Technology Co., Ltd., Beijing 102629, China; ^3^ National Research Center for Veterinary Medicine, Luoyang 471000, China; ^4^ Jiangsu Key Laboratory of Zoonosis, Jiangsu Interdisciplinary Center for Zoonoses and Biosafety, Yangzhou University, Yangzhou 225009, China, yzu.edu.cn

**Keywords:** genetic polymorphism, genotyping, glycosylation pattern, porcine epidemic diarrhea virus (PEDV), recombination

## Abstract

Porcine epidemic diarrhea virus (PEDV) continues to evolve under the selective pressures of vaccine‐induced immunity and environmental factors, leading to immune evasion and sustained piglet mortality. In this study, we characterized PEDV field strains collected across China from 2023 to 2026 and observed substantial genetic divergence. Among them, three strains were identified as the S‐INDEL subtype, with each located on a distinct branch of the S‐INDEL phylogenetic tree. Further analysis revealed branch‐specific molecular features. Notably, strains on the S‐INDEL‐CH‐1 and S‐INDEL‐CH‐2 branches carry a three‐nucleotide deletion at positions 416–418 in the S gene. To facilitate rapid discrimination of subtypes within the GI genotype and S‐INDEL subtype, we established a hierarchical typing scheme that combines predicted N‐linked glycosylation site polymorphisms (N1193 and N230) with the S‐INDEL‐specific deletion pattern, enabling rapid preliminary discrimination among PEDV GI genotype (GIa and GIb) and S‐INDEL subtype directly from S gene sequences. Collectively, our findings provide a complementary and practical framework for PEDV GI genotype and S‐INDEL subtype classification and yield new insights into the molecular characteristics of the S‐INDEL subtype.

## 1. Introduction

The mutation and evolution of viruses and the defense mechanisms of hosts have long been in a state of “dynamic game” and “arms race” [1, 2]. Elucidating the mechanisms that drive such adaptive evolution is a central goal of viral evolutionary biology, a discipline that integrates insights from viral genomics, epidemiology, and host biology [3]. Achieving this goal increasingly relies on computational methods for genomic analysis, including phylogenetic inference, genetic variant classification, and recombination detection, which together form the foundation of modern genomic epidemiology [4].

Porcine epidemic diarrhea virus (PEDV), belongs to the genus *Alphacoronavirus*, was first reported in the 1970s in the United Kingdom and Belgium [5]. The pre‐2010 era of PEDV in China was characterized by sporadic, regional outbreaks dominated by classical GIa strains, whose spread was effectively limited by the extensive use of inactivated and attenuated vaccines based on the CV777 strain [6]. A pivotal shift occurred in October 2010 with the emergence of a highly pathogenic GIIa variant in southern China. This strain demonstrated rapid nationwide transmission and high virulence, circumventing existing vaccine immunity and causing 100% mortality in suckling piglets on vaccinated farms [7]. The global threat of PEDV was further underscored in 2013 by the emergence of a highly pathogenic GIIb strain in the United States, which caused significant mortality (10%) in the national herd and disseminated internationally, leading to substantial economic losses [8]. In 2014, new PEDV variants (OH851 and CH/HBQX/10) were identified in the USA and China [9, 10]. These strains, designated S‐INDEL, are hypothesized to have arisen from recombination events among classical and emerging PEDV strains [11]. Compared with the North American prototype strain, S‐INDEL strains harbor characteristic insertions and deletions in the S gene, including a 1‐nt deletion at position 179, an 11‐nt deletion at positions 163–175, a 3‐nt deletion at positions 425–427, and a 6‐nt insertion at positions 497–502. S‐INDEL strains are associated with milder diarrhea and lower piglet mortality under field conditions compared with highly virulent GII strains [9, 11]. Phylogenetically, S‐INDEL forms a distinct monophyletic clade within genotype I and is now recognized as a GI subtype alongside GIa and GIb [12]. Despite the sustained deployment of commercial vaccines targeting both GI and GII strains, recent surveillance in China reveals persistently high PEDV prevalence and incidence, indicating that the virus remains a critical and unresolved challenge for the swine sector [13].

The genome of PEDV is ~28 kb in length and encodes 4 structural proteins (S, E, M, and N), 16 nonstructural proteins (nsp1‐nsp16), and 1 accessory protein (ORF3) [14]. The S glycoprotein is the main virulence factor of PEDV and is composed of an N‐terminal receptor‐binding S1 subunit and a C‐terminal S2 subunit that contains a fusion element [15]. Consequently, the S gene is both a hotspot of genetic variation underlying virulence divergence and a principal contributor to PEDV’s overall genetic diversity [16]. Given its functional importance and high variability, the S gene serves as the primary genetic marker for reconstructing PEDV evolutionary pathways and classifying genetic lineages [17, 18].

A refined genotyping framework for PEDV was established, utilizing defined amino acid polymorphisms at critical sites rather than relying solely on full‐gene sequence divergence. This approach enabled a clearer elucidation of the virus’s evolutionary pathways and population dynamics [19]. In this study, we adopt the phylogenetic classification framework used in recent large‐scale PEDV genotyping studies [14], in which PEDV strains are classified into GIa, GIb, S‐INDEL, GIIa, GIIb, and GIIc. Building on this framework, to further delineate the genetic diversity within the S‐INDEL subtype, we identified several additional critical predicted N‐linked glycosylation sites. These sites enable the rapid differentiation of strains within the S‐INDEL subtype while also providing new insights into the predicted N‐linked glycosylation motifs in the S protein of this group.

## 2. Materials and Methods

### 2.1. Sample Collection and Sequencing

From 2023 to 2026, a total of 25 PEDV‐positive samples were confirmed by RT‐qPCR and subjected to sequencing by Beijing Sino‐science Gene Technology Co., Ltd. (Beijing, China). The detailed information about these clinical samples were provided in Supporting Information 1: Table S1, and the GenBank accession numbers of the 25 sequenced complete PEDV S genes are provided in Supporting Information 1: Table S2.

### 2.2. Comparative Genomic and Site Polymorphism Analysis

All sequences analyzed in this study were retrieved from the NCBI database. The information regarding the sequences utilized for analysis, including GenBank accession numbers, strain, genotype, collection years, and countries of origin are provided in Supporting Information 1: Tables S3–S8. Nucleotide and deduced amino acid sequences were aligned using MAFFT v7.505 [20]. The genetic distance was calculated using the Maximum Composite Likelihood model in MEGAX with default parameters [21]. Putative predicted N‐linked glycosylation sites were predicted using the NetNGlyc‐1.0 server with a threshold of 0.5 [22]. All glycosylation sites discussed herein are computational predictions that have not been experimentally validated.

### 2.3. Phylogenetic Analysis

Phylogenetic trees of PEDV genetic sequences were constructed using the maximum likelihood (ML) method with 1000 bootstrap replicates in IQ‐TREE v3.1.2. The best‐fitting substitution model was identified using ModelFinder based on Bayesian information criterion (BIC) values (GTR + F+R5 for 87 S gene ML tree) [23]. Branches with bootstrap values higher than 70 were regarded as robustly supported [24]. The results of the phylogenetic analysis were visualized using the iTOL v7 tool (https://itol.embl.de/) [25].

### 2.4. Molecular Modeling and Analysis of the S Protein of PEDV

The S amino acid sequence of 16 S gene sequences of PEDV S‐INDEL strains were retrieved from the NCBI database (Supporting Information 1: Table S3), then aligned, and investigated the effect of deletion of S‐INDEL‐CH‐1 and S‐INDEL‐CH‐2 subtype on the structure of PEDV S protein. The predicted tertiary structures of S region were modeled using the open‐source modeling server SWISS‐MODEL (https://swissmodel.expasy.org/) from the Swiss Institute of Bioinformatics [26]. The tertiary structures of the S monomer were generated using the spike protein of PEDV (PDB ID: 7w73.1) as a reference template. The PyMOL software (The PyMOL Molecular Graphics System, Version 1.7.4 Schrödinger, LLC.) was utilized to visualize and compare these modeled tertiary structures.

## 3. Results

### 3.1. Background Information About the Clinical PEDV Samples in This Study

From 2023 to 2026, a total of 25 PEDV‐positive clinical samples were collected from 11 provinces across the country. The sample type tested including intestinal tissue, fecal swab, and stool (Supporting Information 1: Table S1). The monthly distribution showed sampling across multiple seasons, although peak collection occurred in the winter and early spring months, which is consistent with the known seasonal pattern of PEDV outbreaks.

### 3.2. Phylogenetic Characterization on S Gene of PEDV Strains

**Figure 1:**
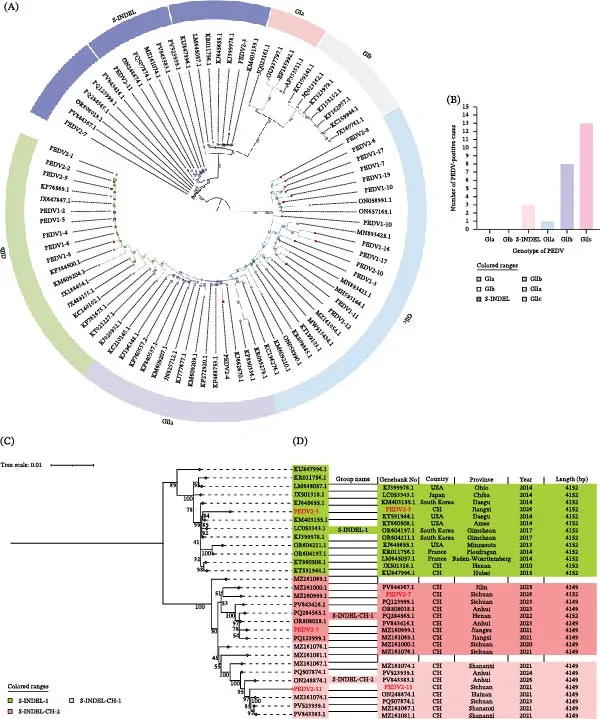
Phylogenetic characterization of PEDV based on S gene. (A) Phylogenetic tree based on the S gene illustrates the genotype distribution of 25 PEDV‐positive clinical samples sequenced in this study and constructed via the maximum‐likelihood method in IQ‐tree software with 1000 bootstrap replicates. Red circles indicate clinical samples analyzed in this study, while other branches represent reference strains from the database. (B) Genotype distribution of PEDV‐positive samples. (C) Phylogenetic tree based on the S gene illustrates the genotype distribution of 3 S‐INDEL clinical samples sequenced in this study and constructed via the maximum‐likelihood method in IQ‐tree software with 1000 bootstrap replicates. (D) Strains information of S‐INDEL.

To investigate the genetic diversity and evolutionary dynamics of circulating PEDV, the PEDV clinical samples were subjected to phylogenetic analysis. Phylogenetic analysis of the S gene revealed distinct genotype distributions, with GIIc being the predominant lineage (56%, 13/25), followed by GIIb (24%, 8/25) and S‐INDEL (12%, 3/25), indicating a regional shift in circulating PEDV from GIIa to GIIc in China (Figure 1A,B). Notably, strains of the S‐INDEL subtype are distributed across two major branches on the phylogenetic tree. To investigate the reasons behind the formation of distinct branches among S‐INDEL PEDV strains, we constructed a separate phylogenetic tree using the S gene sequences of the S‐INDEL subtype and compiled the metadata of all sequences (Figure 1C). Within the phylogenetic tree, the S‐INDEL genotype diverges into two independent clades. The clade containing Chinese strains further separates into two sub‐clades. In this study, we designated these clades as S‐INDEL‐1, S‐INDEL‐CH‐1, and S‐INDEL‐CH‐2. The S‐INDEL‐1 clade comprises strains originating from the USA, South Korea, France, and China (before 2016), all featuring a consistent S gene length of 4152 bp. The clinical strain PEDV2‐3 investigated in this study clusters within this clade. The S‐INDEL‐CH‐1 clade exclusively contains Chinese‐origin strains, with S gene lengths of either 4149 or 4152 bp. Our clinical strain PEDV2‐7 belongs to this clade. Similarly, the S‐INDEL‐CH‐2 clade consists solely of Chinese strains, all possessing an S gene length of 4149 bp, and includes our clinical strain PEDV2‐11. Notably, the S‐INDEL‐1 clade is predominantly composed of strains isolated historically (primarily in earlier years) from both within and outside China. In contrast, the S‐INDEL‐CH‐1 and S‐INDEL‐CH‐2 clades contain exclusively Chinese PEDV strains, all of which are S‐INDEL strains that have emerged since 2020 (Figure 1D).

### 3.3. A Comparative Analysis of Genetic Distance and Evolutionary Rates Across PEDV Genotypes

To further analyze the reasons why the S‐INDEL subtype forms two independent branches on the phylogenetic tree and clarify the molecular evolutionary characteristics of the S‐INDEL subtype compared with other genotypes of PEDV, we performed a comprehensive analysis of within‐genotype genetic divergence (p‐distance) and root‐to‐tip evolutionary rates for GIa, GIb, GIIa, GIIb, GIIc, and the S‐INDEL subtype, using both whole‐genome alignments and complete S gene sequences (Supporting Information 1: Tables S4–S6). The results revealed that the S‐INDEL subtype exhibited an exceptionally high mean p‐distance (0.3809) at the whole‐genome level, ~25‐fold greater than that of any other genotypes. This reflected its remarkable genomic plasticity and suggested that it may not constitute a monophyletic clade with a single, closely shared common ancestor. GI b was the most conserved (0.0016) among the remaining genotypes (Figure 2A). Furthermore, p‐distance analysis also revealed significant differences in genetic diversity among genotypes at the S gene level (Figure 2B). Notably, the S‐INDEL subtype also displayed the highest mean genetic divergence (0.0254), consistent with the whole‐genome findings, followed by GIIc (0.0233), GIIa (0.0109), and GIb (0.0100).

Figure 2A comparative analysis of genetic divergence and evolutionary rates across PEDV genotypes. (A) Genetic distance (p‐distance) of PEDV whole genomes analyzed by genotype. (B) Genetic distance (p‐distance) of PEDV S gene analyzed by genotype. (C–H) Root‐to‐tip regression analysis of PEDV GIa, GIb, GIIa, GIIb, S‐INDEL, and GIIc subtype (distance versus sampling time).

**Figure:**
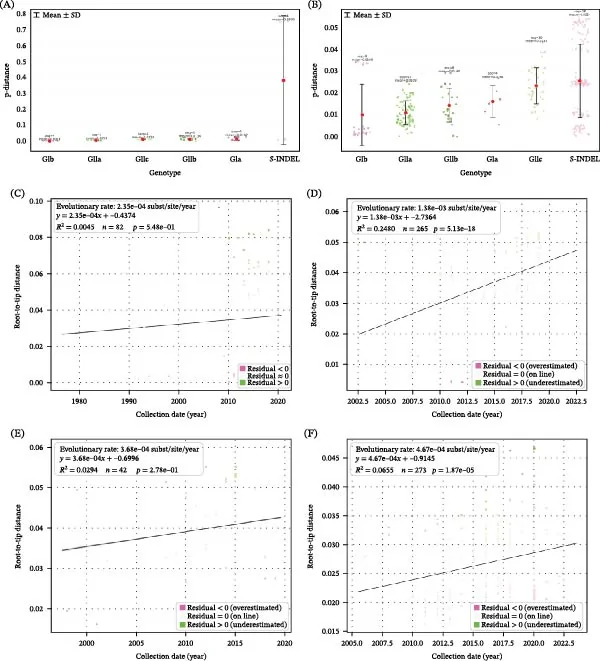

**Figure:**
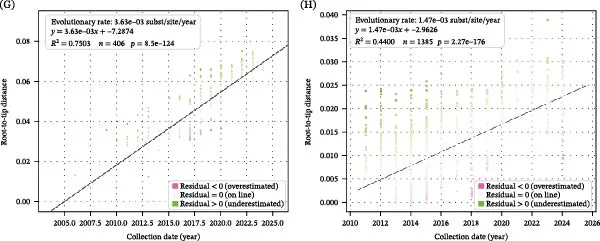

Furthermore, root‐to‐tip regression analysis using TempEst, which correlated estimated genetic distances with sampling dates, demonstrated significant heterogeneity in evolutionary rates among genotypes (Figure 2C–H). The S‐INDEL subtype exhibited the fastest evolutionary rate (3.63 × 10^−3^ substitutions/site/year) with a strong temporal signal (*R* ^2^ = 0.7503), supporting preliminary rate comparison among genotypes. GIIc exhibited the second highest evolutionary rate (1.47 × 10^−3^ substitutions/site/year, *R* ^2^ = 0.4400). In contrast, GIIa (3.68 × 10^−4^ substitutions/site/year, *R* ^2^ = 0.0294) and GI a (2.35 × 10^−4^ substitutions/site/year, *R* ^2^ = 0.0045) showed weak temporal signals. This may indicate either insufficient temporal sampling or nucleotide substitution saturation in these more anciently diversified genotypes.

The above findings delineate the distinct evolutionary trajectories of PEDV genotypes. Notably, the S‐INDEL subtype demonstrates the highest genetic divergence at both genomic and S‐gene levels, representing the most plastic PEDV lineage, and concurrently exhibits the fastest evolutionary rate, underscoring its significant clinical importance.

### 3.4. Analysis of Amino Acid Differences of S Protein in PEDV Clinical Strains

Given that the S protein is the principal target of host neutralizing antibodies, we compared the amino acid similarity of clinical strains with three reference strains representing distinct genotypes: the GIa vaccine strain CV777, the GIIb epidemic strain AJ1102, and a representative GIIc strain (the predominant genotype in this study, 56%). The clinical strains showed 92.6%–96.1% similarity with CV777, 95.0%–99.9% with AJ1102, and 95.5%–99.3% with the GIIc reference strain CH/HNAY/2015 (Figure 3A–C). Domain‐resolution analysis revealed that amino acid variation was primarily concentrated in the D0 and NTD domains of the S1 subunit across all three comparisons (Figure 3D–F), with strains PEDV2‐3, PEDV2‐7, and PEDV2‐11 exhibiting the most pronounced divergence in these N‐terminal regions. We further mapped the distribution of amino acid substitutions relative to known neutralizing epitope regions on the S protein domain architecture, including COE (aa 499–638), SS2 (aa 748–755), SS6 (aa 764–771), and the S1‐NTD antigenic region (aa 1–400) (Figure 3D–F). The D0 and NTD domains, which encompass the S1‐NTD antigenic region, harbored the highest density of substitutions, whereas the COE, SS2, and SS6 epitopes were relatively conserved among clinical strains. Consistently, the major neutralizing epitopes COE, SS2, SS6, 2C10, and S1A are 100% conserved between S‐INDEL and GII, with substitutions restricted to the N‐terminal S10/D0 epitope (Supporting Information 2: Figure S1A, B).

Figure 3Analysis of amino acid homology of S protein among PEDV clinical strains and the CV777 or AJ1102 strain. (A–C) Amino acid homology of the S protein of PEDV clinical strains were compared with CV777, AJ1102, and CH/HNAY/2015 strains. (D–F) The amino acid variation regions in the S protein of PEDV clinical strains were compared with CV777, AJ1102, and CH/HNAY/2015 strains.

**Figure:**
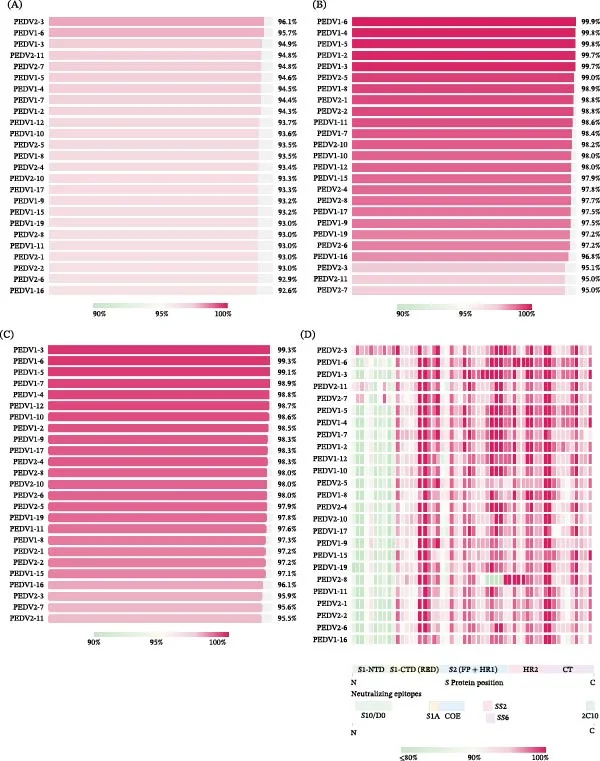

**Figure:**
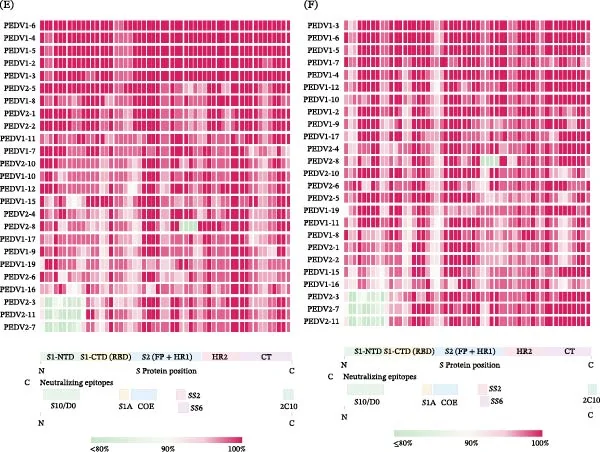

**Figure 4:**
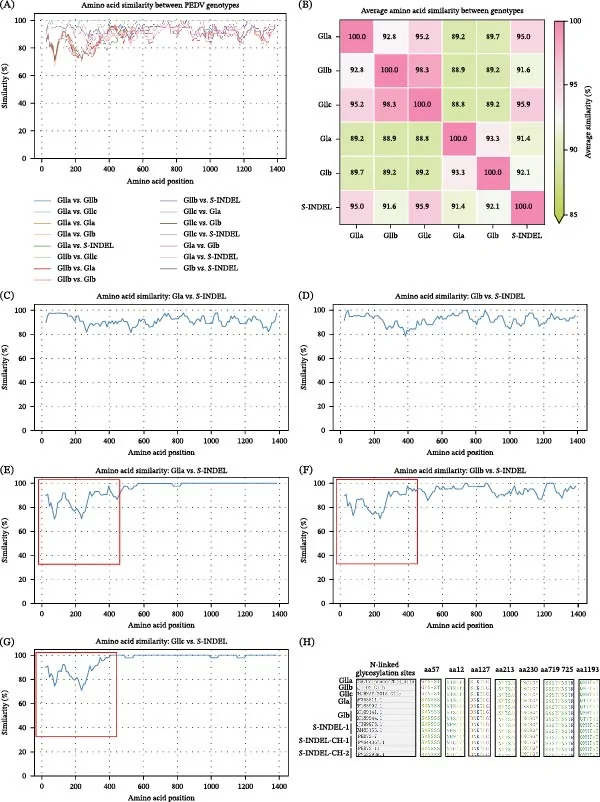
Analysis of S protein amino acid sequence similarity and local divergence among PEDV genotypes. (A,B) Amino acid similarity among PEDV genotypes. (C) Amino acid similarity among GIa and S‐INDEL subtypes. (D) Amino acid similarity among GIb and S‐INDEL subtype. (E) Amino acid similarity among GIIa and S‐INDEL subtypes (Red boxes indicate regions of high sequence divergence). (F) Amino acid similarity among GIIb and S‐INDEL subtypes (Red boxes indicate regions of high sequence divergence). (G) Amino acid similarity among GIIc and S‐INDEL subtypes (Red boxes indicate regions of high sequence divergence). (H) Analysis of N‐linked glycosylation motifs at key amino acid sites between the S‐INDEL and GI/GII genotype.

Furthermore, to analyze the amino acid differences in the S gene among different genotypes, we performed pairwise comparisons of the S gene amino acid sequences. The results showed that significant differences among genotypes were primarily concentrated in the 0–400 aa region (D0 and NTD domain) (Figure 4A). Domain‐wise, D0 was the only divergent domain (85.95% identity), whereas RBD, SD1, SD2, and S2 were 100% identical between the two consensus sequences (Supporting Information 2: Figure S1C, D). Among them, the S‐INDEL subtype exhibited the highest amino acid similarity (95.9%) with GIIc (Figure 4B). Its overall similarity to GIa and GIb was relatively uniform, whereas the major differences compared to GII genotypes were predominantly localized within the D0 region of the S gene (Figure 4C–G). S‐INDEL and GII S proteins differ by 29 amino acid substitutions, 26 of which are confined to the D0 domain (aa 34–234) (Supporting Information 2: Figure S1E, F). Furthermore, S‐INDEL subtypes (S‐INDEL‐1, S‐INDEL‐CH‐1, and S‐INDEL‐CH‐2) exhibit partial sequence conservation with GII genotypes (GIIa, GIIb, and GIIc) at most predicted linked N‐glycosylation sites (aa719/725, aa1193) but harbor unique substitutions at key positions (aa57, aa112, and aa127; Figure 4H). In summary, these findings delineated that PEDV clinical strains demonstrated higher genetic similarity to the vaccine strain AJ1102 than to CV777, particularly within the antigenically critical S gene. Amino acid variation among genotypes was predominantly localized to the N‐terminal D0 and NTD domain (aa 0–400) of the S protein, with the S‐INDEL subtype showing the greatest homology to GIIc within this region and showing different predicted N‐linked glycosylation sites with the GII genotype.

### 3.5. Analysis of Deletion and Insertion Patterns in the S Gene of PEDV S‐INDEL Strains

The characteristic features of the S‐INDEL strains include a 1‐nucleotide deletion at 179 nt, an 11‐nucleotide deletion at 163–175 nt, a 3‐nucleotide deletion at 425–427 nt, and a 6‐nucleotide insertion at 497–502 nt [27]. Our alignment revealed that all S‐INDEL strains in this study harbor these characteristic deletion/insertion patterns (Figure 5A). At the residue level, these N‐terminal indels are GII—specific‐the IGENQ (aa 54–58) and N (aa 139) insertions and deletions at aa 58/156/157—whereas S‐INDEL and classical GI share an identical indel pattern (Supporting Information 2: Figure S2A, B). Notably, the alignment mapping of nucleotide on S‐INDEL‐CH‐1/CH‐2 strains exhibit a 3‐nucleotide deletion fragment at positions 416–418 in the S gene (Figure 5A). Amino acid alignment of this region indicated that the deletion results in its replacement with the motif “PSSG” (Figure 5B). Subsequently, to investigate if the deletion of S‐INDEL‐CH‐1/CH‐2 altered the surface structure of the S protein, we generated the 3D models of the S protein (PDB ID: 7w73.1.A) as a template for modeling (Figure 5C). The findings revealed that the S‐INDEL‐CH‐1/CH‐2 gene deletion changed the top region of the S1 protein surface structure, compare to the OH851 (KJ399978.1; Figure 5D,E). Homology modeling suggested that the 416–418 nt deletion may affect the local surface topology of the S1 region, but this requires experimental validation.

Figure 5Analysis of deletion and insertion patterns in the S gene of PEDV S‐INDEL subtype. (A) Comparison of nucleotide insertion and deletion patterns between the S‐INDEL subtype and the USA/Colorado/2013 strain. (B) Comparison of amino acid insertion and deletion patterns between the S‐INDEL subtype and the USA/Colorado/2013 strain. (C–E) Comparative analysis of the predicted S protein modeling between the new deletion sequence of USA/Colorado/2013 strain, OH851 strain, and S‐INDEL‐CH‐1/CH‐2 subtype.

**Figure:**
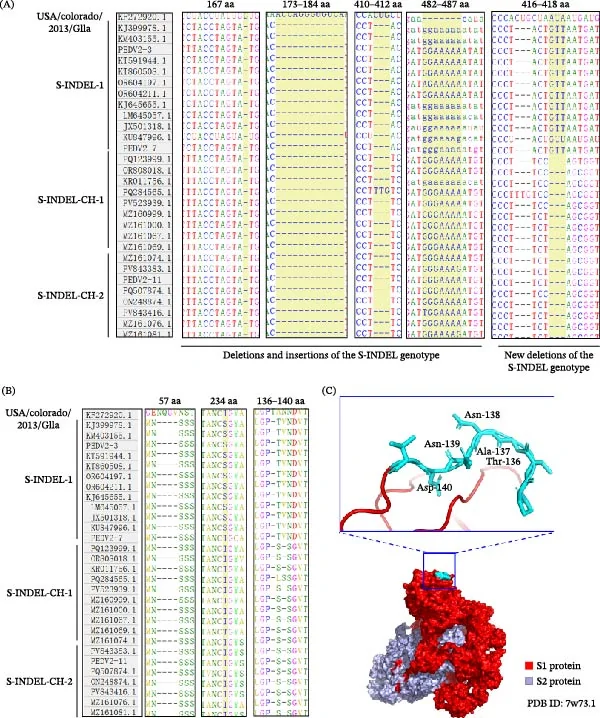

**Figure:**
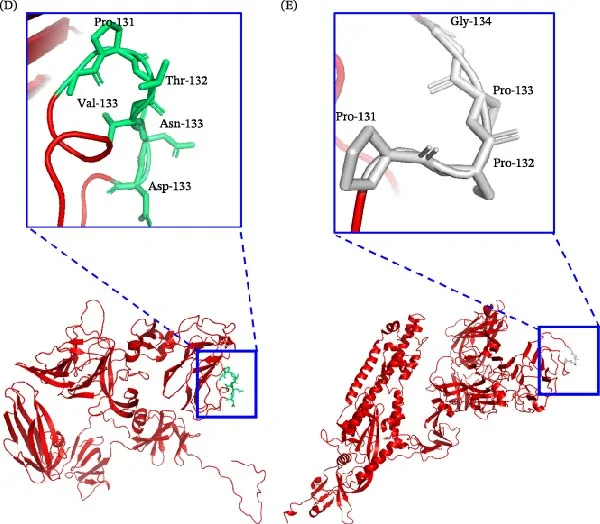

### 3.6. Analysis of Glycosylation Patterns in PEDV GI Genotype

**Figure 6:**
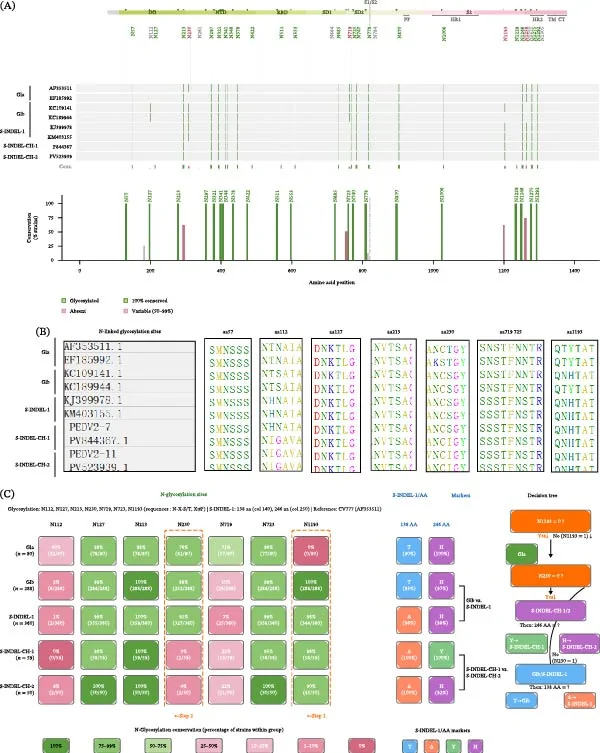
Analysis of glycosylation patterns in PEDV GI genotype. (A) Analysis of glycosylation patterns in PEDV GI genotype with reference strain. (B) Analysis of glycosylation motifs at key amino acid sites in the GI genotype. (C) Distribution of S protein glycosylation patterns across PEDV subtypes.

To further investigate the determinant sites of PEDV GI genotyping, we primarily selected 8 reference strains with distinct backgrounds in GI genotype to predict N‐glycosylation sites on the S protein. A total of 31 putative predicted N‐linked glycosylation sites (N57‐N1305) were identified on the S protein across the 8 PEDV strains examined (Supporting Information 1: Table S7). Specifically, 21 sites showed absolute conservation (Figure 6A). Next, we selected 13 reference strains covering GIa, GIb, S‐INDEL, GIIa, GIIb, and GIIc to predict N‐glycosylation sites on the S protein. Between the S‐INDEL and GII consensus sets, 27 of the predicted N‐glycosylation sites are shared. S‐INDEL carries lineage‐specific N127 and N230, whereas GII carries N114 (Supporting Information 2: Figure S2C). We selected sites reported in the literature to be associated with PEDV virulence for analysis [28]. N57, N127, and N213 in the D0 domain were conserved as the NSS, NKT, and NVT motif across all GI genotypes. N112 in the D0 domain was predominantly located in the GI subtype. N230 in the NTD domain was predominantly located in the GIa, GIb, and S‐INDEL‐1 subtypes. N719 and N723 in the SD2 domain, located near the S1/S2 cleavage site, requires the presence of two consecutive NXT motifs from positions 719 to 725 in the GIa and GIb subtypes (where X presents any amino acid except proline or asparagine). And N1193 was predominantly located in the S‐INDEL subtype (Figure 6B).

Furthermore, we downloaded all available S gene sequences of the GIa, GIb, and S‐INDEL subtypes from the NCBI database (Supporting Information 1: Table S8). The glycosylation patterns at key sites were statistically analyzed for each subtype. The results revealed that N‐glycosylation at positions 1193 and 230 (N1193 and N230) exhibited distinct polymorphism patterns. We therefore established a hierarchical decision tree for GI genotype classification (Figure 6C). Firstly, GIa can be separated from all other subtypes by the absence of the N1193 glycosylation site (0%, 0/80), whereas GIb, S‐INDEL‐1, S‐INDEL‐CH‐1, and S‐INDEL‐CH‐2 uniformly harbor N1193 (≥90%). Secondly, among N1193‐positive strains, S‐INDEL‐CH‐1 and S‐INDEL‐CH‐2 can be distinguished from GIb and S‐INDEL‐1 by the near‐complete absence of the N230 glycosylation site (3% and 4%, respectively), whereas GIb and S‐INDEL‐1 retain N230 (88% and 91%). Finally, the remaining pairs are resolved by amino acid markers—GIb (138 aa = *T*, 90%) is distinguished from S‐INDEL‐1 (138 aa = *Δ*, 96%), while S‐INDEL‐CH‐1 (246 aa = *Y*, 100%) is distinguished from S‐INDEL‐CH‐2 (246 aa = *H*, 92%). In summary, we established a hierarchical molecular typing scheme for GI genotypes that combines predicted N‐linked glycosylation site polymorphisms (N1193 and N230) with amino acid markers at positions 138 and 246.

## 4. Discussion

PEDV remains a significant concern that affects the development of healthy animals in the pork industry worldwide [29]. Immunization with safe and effective vaccines and biosecurity measures remain the main approach for controlling the spread of PEDV. However, the available vaccines cannot provide complete protection against newly emerging virulent strains of PEDV, which may result in recurrent outbreaks. The main reason for this is the high degree of variation in the S protein [30]. Moreover, this high level of diversity has persisted since the outbreak in China in 2010, posing a significant challenge to the prevention and control of PED [6]. In this study, we conducted a 3‐year investigation of PEDV in China and found high genetic diversity in the S protein among the prevailing strains, which aligns with findings from previous studies [19, 27]. Currently, the GIIc genotype remains dominant (56%, 14/25). However, it is noteworthy that three strains of the S‐INDEL subtype were detected (12%, 3/25). Phylogenetic analysis revealed that these three S‐INDEL strains are located on three distinct minor clades in this study.

The term S‐INDEL was coined for novel PEDV variants (OH851 and CH/HBZX/10) reported in the USA and China in 2014, which are defined by three deletions and one insertion in comparison with the North American prototype strain [9, 10]. S‐INDEL strains are thought to be associated with reduced severity of clinical disease in pigs under field conditions [9, 11]. We further analyzed the background information of these three clades and found that S‐INDEL‐1 clade is predominantly composed of strains isolated historically (primarily in earlier years) from both within and outside China. In contrast, the S‐INDEL‐CH‐1 and S‐INDEL‐CH‐2 clades contain exclusively Chinese PEDV strains, all of which are S‐INDEL strains that have emerged since 2020. Furthermore, the nucleotide length of the S gene also exhibits a discernible pattern across different phylogenetic branches. Additionally, we conducted a comparative analysis of the S gene sequences between the S‐INDEL subtype identified in this study and the classical S‐INDEL strain OH851. The analysis revealed that the S‐INDEL‐CH‐1/CH‐2 subtypes possess an additional three‐nucleotide deletion (GTT/GUU) at positions 416–418. Subsequently, utilizing 3D models of the S protein (PDB ID: 7w73.1.A) as a template for modeling, we discovered that S‐INDEL‐CH‐1/CH‐2 gene deletion changed the top region of the S1 protein surface structure, compared to the OH851 (KJ399978.1), which may indicate a new mutation pattern of PEDV. Genetic distance and evolutionary rate serve as complementary metrics, providing quantitative evidence for resolving genetic divergence between species [31]. Additionally, we compared the genetic divergence and evolutionary rates across PEDV genotypes, found that the S‐INDEL subtype exhibited the highest mean genetic divergence (0.0254) consistent with the whole‐genome findings (0.3809), and it also exhibited the fastest evolutionary rate (3.63 × 10^−3^ substitutions/site/year) with a highly significant temporal signal (*R* ^2^ = 0.7503). Therefore, monitoring the epidemic dynamics of PEDV S‐INDEL subtypes is essential for timely intervention, while elucidating their evolutionary pathways informs the development of sustainable control measures.

The spike protein of PEDV has extensive predicted N‐linked glycosylation sites on its surface that are involved in conformational regulation and immune evasion [28, 32]. Previous studies have documented the link between the glycosylation motifs and pathogenicity. The virulence of the recombinant strains (rPEDV‐Smut62 [57ENQGVNST64 to 57NSSS60 modification], rPEDV‐Smut118 [116NTNA119 to 116NTSA119 modification], rPEDV‐Smut131 [131IKTL134 to 131NKTL134 modification], rPEDV‐Smut235 [233NCIG236 to 233NCSG236 modification], and rPEDV‐Smut722 [722SSTF725 to 722NSTF725 modification]) were lower than rPEDV‐S_wt_ strain. Furthermore, recombinant strains rPEDV‐Smut62 and rPEDV‐Smut722 were capable of inducing cross‐protective immunity in piglets. However, rPEDV‐Smut235 was not successfully rescued [29]. The N‐glycan of N1193, situated on the S2 subunit, connects the protein fusion regions HR1 and HR2 (CD domain), it is essential for mediating the fusion between the viral envelope and the host cell membrane during viral entry [33]. The glycosylation patterns at these mutation sites were also analyzed among different subtypes of the GI genotype and S‐INDEL subtype in this study. Our analysis revealed distinct polymorphism patterns specific to the GI genotype and S‐INDEL subtype. The S‐INDEL dataset (*N* = 469) enabled a comprehensive assessment of predicted glycosylation site polymorphisms across S‐INDEL subtype. We established a hierarchical typing scheme based on two predicted glycosylation sites (N1193 and N230) and two amino acid markers (positions 138 and 246) that together resolve GI genotype and S‐INDEL subtype in a three‐step decision tree (Figure 6C). The use of N1193 to distinguish GIa from GIb is consistent with Lei et al. [19], we extend this framework by incorporating N230—whose loss specifically marks the emerging Chinese S‐INDEL‐CH‐1/CH‐2 lineages and amino acid markers at positions 138 and 246 for fine‐resolution subtyping. This combined approach, validated on a large‐scale dataset, provides a robust typing tool for PEDV GI genotypes and S‐INDEL subtype.

Li et al. [34] recently classified S‐INDEL strains into two major clades—early endemic (Clade 1) and recently dominant Chinese strains (Clade 2)—and identified 22 clade‐specific amino acid substitutions, 11 of which are located in the D0 domain. This is consistent with our observation that the D0 domain is the primary region of amino acid divergence across genotypes [34]. Both studies recognize a fundamental split between historical globally distributed strains and recently emergent Chinese strains. Our analysis further identifies a 416–418 deletion within the Chinese lineage. Our analysis further reveals a 416–418 nt deletion within Chinese S‐INDEL isolates, which is consistent with the observation by Li et al. [34] that a deletion of 314 amino acids is present in Clade 2 of S‐INDEL strains. And resolves it into two sub‐branches (S‐INDEL‐CH‐1 and S‐INDEL‐CH‐2) based on S gene length polymorphism and amino acid markers (position 246). However, Li et al. [34] did not discuss the variation at amino acid position 24 in Clade 1 and Clade 2 of S‐INDEL strains in their study. While Li et al. [34] employed phylogeographic and recombination analyses, our glycosylation‐based hierarchical typing scheme offers a complementary, sequence‐based tool for rapid genotype screening in surveillance settings. Fu et al. [12] reported that among GIIb recombinant strains, the majority were recombinants between different GIIb strains (M609204.1, KU646831.1, and MZ161041.1), while a small number of GIIb recombinant strains were formed by recombination between GIIb strains and S‐INDEL strains, with the recombination region containing HR1.

We acknowledge that this study has some potential limitations. For instance, our collection of full‐length PEDV genomes from 2023 to 2026 was limited in both sample size and provinces. Consequently, the data may not provide a whole of PEDV epidemiology across China during these years. However, to enhance the representativeness of our analysis on the S‐INDEL subtype, we have downloaded all currently S‐INDEL S gene sequences from the NCBI database. The patterns identified from this comprehensive dataset provide novel insights into the S‐INDEL subtype, including the patterns of glycosylation motifs at key sites and deletion patterns. In addition, we identified a three‐nucleotide deletion (positions 416–418) in the S‐INDEL‐CH‐1/CH‐2 subtype, and current functional analysis was restricted to 3D modeling of the S protein, as opposed to experimental validation of the recombination strain’s biological properties in cellular or piglet models [28]. The association between genotypes/INDEL patterns and pathogenicity as well as cross‐neutralization potential warrant future investigation. Furthermore, the glycosylation‐based typing scheme is intended as a rapid screening tool for molecular surveillance and has been validated only on currently available sequences; borderline or discordant cases should be resolved by complete S gene sequencing and ML phylogenetic reconstruction, and its performance on future emerging variants require ongoing evaluation.

## 5. Conclusion

In summary, in this study, we identified a 3‐nucleotide deletion within the S gene of the S‐INDEL‐CH‐1/CH‐2 subcluster, further underscoring the ongoing diversification of this evolutionarily dynamic lineage. Additionally, we established a hierarchical typing scheme that combines predicted N‐linked glycosylation site polymorphisms (N1193 and N230) with the S‐INDEL‐specific deletion pattern, enabling rapid preliminary discrimination among PEDV GI genotype (GIa and GIb) and S‐INDEL subtype directly from S gene sequences. This approach provides a complementary tool that supplements, rather than replaces, comprehensive phylogenetic analysis and offers a practical method for preliminary genotype screening in large‐scale molecular surveillance. These findings extend the current genotyping framework for PEDV and provide actionable molecular markers for monitoring the evolution of the GI genotype and S‐INDEL subtype.

## Author Contributions

**Yue Chen** and **Lin Yuan:** investigation, data curation, writing – original draft. **Wenqiang Wang, Lulu Chen, Can Cui, Jie Zhang, Zhenbang Zhu**, and **Wei Wen:** writing – review and editing. **Kegong Tian** and **Xiangdong Li:** conceptualization, funding acquisition, supervision.

## Funding

This work was supported by the National Key Research and Development Program of China (Grant 2023YFD1800500), the Postgraduate Research & Practice Innovation Program of Jiangsu Province (Grant 26CXJH6960), the Taishan Industrial Leading Talent Project (Grant tscx202507154), the 111 Project D18007, and the Priority Academic Program Development of Jiangsu Higher Education Institutions (PAPD).

## Disclosure

All authors have read and approved the final manuscript.

## Ethics Statement

This study involved analyses of viral sequences obtained from metagenomic sequencing data and publicly available genomic datasets retrieved from established databases. No human participants, animals, or identifiable personal information were directly involved, and no experimental procedures were performed on living subjects in this study. Therefore, ethical approval and informed consent were not required.

## Conflicts of Interest

The authors declare no conflicts of interest.

## Supporting Information

Additional supporting information can be found online in the Supporting Information section.

## Supporting information


**Supporting Information 1** Table S1: Background information of PEDV‐positive sample (2023−2026). Table S2: Information of strains sequenced in this study. Table S3: Reference strains for constructing the phylogenetic tree based on the S gene. Table S4: Reference strains for analyzing genetic distance base on whole‐genome. Table S5: Reference strains for analyzing genetic distance based on S gene. Table S6: Reference strains for analyzing evolutionary rates base on S gene. Table S7: Representative strains of each PEDV GI genotype and S‐INDEL subtype for N‐glycosylation site analysis. Table S8: Statistical analysis of glycosylation sites in PEDV genotypes GIa, GIb, and S‐INDEL.

**Supporting Information 2** Figure S1: Comparing neutralizing epitopes, functional domains, and amino acid substitutions in the S proteins of S‐INDEL and GII strains. (A) Neutralizing epitopes along the S protein. (B) Consensus identity within each epitope in the S proteins of S‐INDEL and GII strains. (C) Functional‐domain architecture of the PEDV S protein. (D) Consensus identity per functional domain in the S proteins of S‐INDEL and GII strains. (E) Positions of the 29 substitutions in the S protein. (F) The names of substituted amino acids at each site. Figure S2: Comparing indel patterns and glycosylation sites in the S proteins of S‐INDEL and GII strains. (A, B) Insertion and deletion map in the S proteins of S‐INDEL and GII strains. (C) Comparing glycosylation sites in the S proteins of S‐INDEL and GII strains.

## Data Availability

The data that support the findings of this study are available from the corresponding author upon reasonable request.
