# Supplementary material for: Phylogenetic Divergence and Molecular Signatures of Porcine Epidemic Diarrhea Virus S‐INDEL Subtype
Source: Transbound Emerg Dis. 2026 Sep 12;2026:1604677. doi: 10.1155/tbed/1604677 (PMC13570654; doi:10.1155/tbed/1604677)
Supplement: Supplementary file 1 [file TBED-2026-1604677-s002.docx]

**FIGURE S1**

**FIGURE S1** Comparing neutralizing epitopes, functional domains, and amino acid substitutions in the S proteins of S-INDEL and GII strains. **(A)** Neutralizing epitopes along the S protein. **(B)** Consensus identity within each epitope in the S proteins of S-INDEL and GII strains. **(C)** Functional-domain architecture of the PEDV S protein. **(D)** Consensus identity per functional domain in the S proteins of S-INDEL and GII strains. **(E)** Positions of the 29 substitutions in the S protein. **(F)** The names of substituted amino acids at each site.

**FIGURE S2**

**FIGURE S2** Comparing indel patterns and glycosylation sites in the S proteins of S-INDEL and GII strains. **(A, B)** Insertion and deletion map in the S proteins of S-INDEL and GII strains. **(C)** Comparing glycosylation sites in the S proteins of S-INDEL and GII strains.
